# Fruitful Pregnancy Outcome in a Case of Eisenmenger Syndrome With Severe Pulmonary Hypertension: A Rare Case Report

**DOI:** 10.7759/cureus.21068

**Published:** 2022-01-10

**Authors:** Faten Alsomali, Shahida Mushtaq, Mohamad Bakir, Sami Almustanyir

**Affiliations:** 1 Medicine, Alfaisal University, Riyadh, SAU; 2 Obstetrics and Gynecology, King Faisal Specialist Hospital and Research Centre, Riyadh, SAU

**Keywords:** pulmonary hypertension, pregnancy, multidisciplinary decision-making, atrial septal defect secundum, eisenmenger syndrome

## Abstract

Eisenmenger syndrome (ES) is considered an absolute contraindication for pregnancy. ES is characterized by a congenital heart abnormality that results in a significant anatomical shunt. Hemodynamic forces generate a left-right shunt, leading to severe pulmonary arterial hypertension (PAH). Eventually, the shunt will become a right-to-left shunt due to increased pulmonary vascular resistance, leading to significant hypoxemia and cyanosis. Pregnant women with ES experience volume overload as a result of the syndrome and the physiological response of pregnancy. The decrease in systemic vascular resistance that occurs during pregnancy also increases the right-to-left shunt, resulting in left ventricular failure. Due to the significant risk to both the mother and the fetus, women are advised to terminate their pregnancy during the first trimester. However, with all the odds, very few cases show positive neonatal and maternal outcomes. Appropriate management of ES includes a multidisciplinary team assembled to monitor and manage the patient carefully and thoroughly. In this paper, we present a case of ES secondary to an atrial septal defect with severe PAH in a 32-year-old woman who underwent a cesarean section at 33 weeks of gestation. She delivered a healthy baby girl. On the seventh postoperative day, she was discharged with no complications.

## Introduction

Eisenmenger syndrome (ES) is a group of symptoms caused by a congenital heart defect that leads to a large anatomic shunt [1]. Because of structural variations found at birth, hemodynamic forces cause a left-right shunt, which progresses to severe pulmonary arterial hypertension (PAH) and high vascular resistance. The left-to-right shunt will eventually become a right-to-left shunt due to increased pulmonary vascular resistance, leading to substantial hypoxemia and cyanosis [1]. PAH is defined as a mean pulmonary arterial pressure of more than 25 mmHg at rest or 30 mmHg while exercising. This can happen as early as the first decade of life in cases with massive shunts or severe, unrepaired congenital cardiac disease [1]. ES is a relatively rare condition that is typically encountered in people with little access to healthcare (i.e., in remote and underserved regions), where large anatomical anomalies can go unnoticed for many years [1]. A patient with established congenital heart disease (CHD) who presents with progressive exertional dyspnea is the most prevalent presentation of ES. Volume retention, swelling, syncope, palpitations, cyanosis, or hemoptysis are all typical symptoms. In addition, patients may experience polycythemia and viscosity symptoms such as headaches, dizziness, visual abnormalities, stroke, and end-organ damage as a result of the increase in red blood cell volume caused by continuous hypoxia [1]. Pregnancy-related hemodynamic alterations in ES are poorly tolerated, putting patients at risk of quickly escalating cardiac decompensation, thrombotic events, and abrupt demise [2]. In this paper, we present a case of ES secondary to an atrial septal defect with severe PAH in a 32-year-old pregnant woman.

## Case presentation

Our patient is a 32-year-old woman, gravida two para one with no previous miscarriages or stillbirths (G2 P1+0). She is a known case of ES secondary to atrial septal defect with severe PAH.

In her first pregnancy, she underwent a cesarean section at 34 weeks of gestation due to ES and severe PAH (PAH). She was advised not to conceive again and she was also counseled to terminate her current pregnancy while she was in her first trimester but she refused. She has a negative family history of CHD. She was on phosphodiesterase 5 (PDE5) inhibitor (sildenafil), synthetic prostacyclin (prostaglandin I2 [PGI2]) analogs (iloprost), and enoxaparin sodium (Clexane) but was not compliant with her medications. She is on home oxygen therapy of 3 liters via nasal cannula.

She was admitted at 28 weeks gestation with the complaint of shortness of breath and chest pain of grabbing nature that increased on exertion. The patient’s saturation on room air at the time of presentation was 88%.

On general examination, she looked ill and pale. She had dry mucus membranes and mild pallor of the oral mucosa. Her vital signs showed low blood pressure. The patient had low oxygen saturation. Her heart rate and respiratory rate were within normal values as shown in Table 1.

**Table 1:** Vital signs of our patient in comparison to normal values.

Vital sign	Patient’s result	Reference range
Temperature	36.8°C	36.6°C to 37°C
Heart rate	88 beats per minute	60-100 beats per minute
Respiratory rate	18 breaths per minute	16-20 breaths per minute
Blood pressure	95/60 mmHg	120/80 mmHg
Oxygen saturation	88% on room air	95%-100% on room air
Body mass index	25.3 kg/m^2^	18.5 kg/m^2^ to 24.9 kg/m^2^

On respiratory examination, there were bilateral vesicular breath sounds on all lung fields. On cardiovascular examination, jugular venous pressure (JVP) was normal not exceeding 3-4 cm above the sternal angle. On auscultation, there was a normal first heart sound (S1). There was a fixed split in the second heart sound (S2) with a systolic murmur in the pulmonic area.

**Figure 1:**
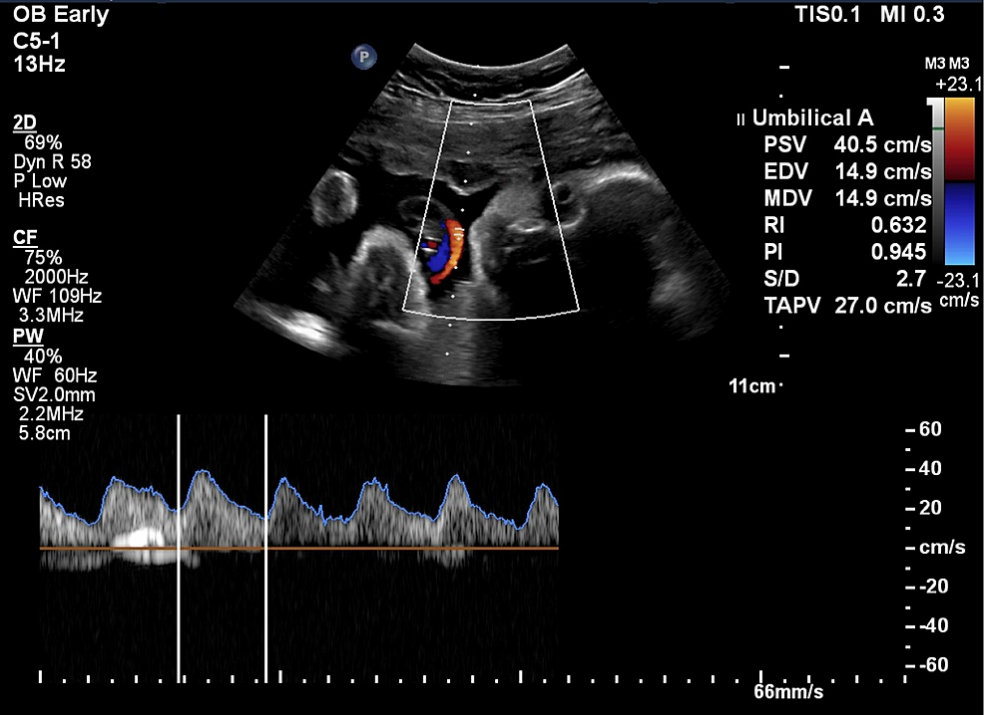
Umbilical artery Doppler ultrasound for a singleton pregnancy at 28 weeks gestation reveals normal values.

On abdominal examination, her uterus corresponded to 28-week gestation in size. Abdominal ultrasound showed a single viable fetus in cephalic presentation. Abdominal circumference was in the 10% percentile. The fetus weighed 1456 g. The amniotic index was in the normal range and the deepest pool was 2.7 cm. Placenta was posterior and high. Doppler ultrasound (Figure 1) showed a normal umbilical artery pulsatility index as shown in Table 2.

**Table 2:** Patient’s umbilical artery Ultrasound values in comparison to the normal range. As demonstrated in Figure 1.

Umbilical artery ultrasound value	Patient’s result	Reference range
The amniotic index	8.4 cm	5 cm to 25 cm
Umbilical artery pulsatility index	0.95	1.24 with a standard deviation of +/- 0.27

The vaginal examination showed a posterior high cervix, uneffaced, firm and closed internal os.

Laboratory investigations revealed low hemoglobin. The patient’s hematocrit was on the lower end of the normal range, and otherwise normal complete blood count (CBC). Renal profile and electrolytes were in the normal range as shown in Table 3.

**Table 3:** Laboratory investigations of our patient in comparison to normal values.

Laboratory test	Patient’s result	Reference range
Hemoglobin	11.8 g/dL	12-15 g/dL (women)
Hematocrit	38%	36%-47% (women)
Sodium	138 mmol/L	135-147 mmol/L
Potassium	3.7 mmol/L	3.5-5.0 mmol/L
Chloride	100 mmol/L	98-106 mmol/L
Urea level	3.6 mmol/L	3.6-7.1 mmol/L
Creatinine	55 μmol/L	44-97 μmol/L
Estimated glomerular filtration rate (eGFR)	> 60 mL/min/1.73 m^2^	119.2 mL/min/1.73m^2^

**Figure 2:**
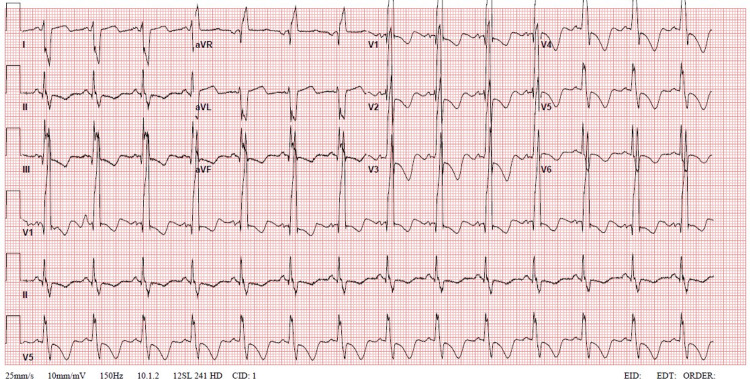
ECG demonstrates normal sinus rhythm, right axis deviation, right bundle branch block, and T wave inversion in leads V1-V3. ECG: echocardiogram

Her most recent echocardiogram (ECG) showed normal sinus rhythm, right axis deviation, right bundle branch block, and T wave inversion in leads V1-V3 (Figure 2).

**Figure 3:**
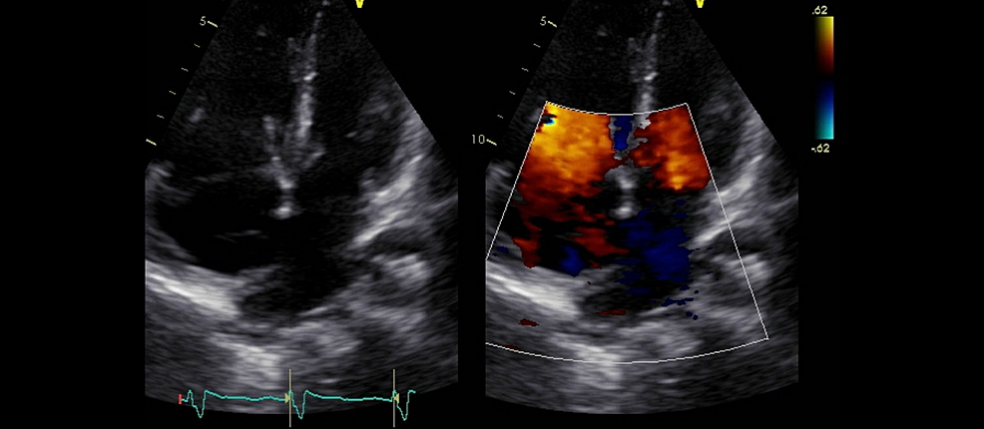
Transthoracic echocardiogram demonstrates a large secundum atrial septal defect with bidirectional interatrial shunt, predominately from left to right. There is moderate right atrial enlargement.

Echo demonstrated severely elevated right ventricular pressure of 73 mmHg (normal range: systolic pressure below 30 mmHg). A large secundum atrial septal defect with bidirectional interatrial shunt, predominately from left to right was observed. There is moderate right atrial enlargement. The pulmonic valve is thin and pliable with mild pulmonic valvular regurgitation and severe pulmonary artery dilation. There is also mild tricuspid regurgitation. Systolic pulmonary arterial pressure is greater than or equal to 60 mmHg (Figure 3).

She was discharged after being on 5 liters of oxygen via nasal cannula, and her oxygen saturation was at her target of greater than 88%. At 30 weeks gestation during her final antenatal visit, she reported good fetal movements and had a reactive non-stress test with good variability. During a bedside ultrasound, the estimated fetal weight was 1700 g. It also showed a normal amniotic index and umbilical artery pulsatility index of 1.4. She received her first dose of betamethasone, and she was given a plan of delivery at 33 weeks of gestation after a discussion at a multidisciplinary meeting.

She was admitted for an elective low transverse cesarean section at 33 weeks of gestation under epidural anesthesia using fentanyl 2 mcg/mL and bupivacaine 0.1%. Due to her high-risk pregnancy, a cardiologist, cardiac and obstetric anesthesiologists, and extracorporeal membrane oxygenation (ECMO) team were on standby.

She gave birth to a healthy female infant. She had an Apgar (Appearance, Pulse, Grimace, Activity, and Respiration) score of 5 at 1 minute, 7 at 5 minutes, and 9 at 10 minutes (normal range: Apgar score of 7 and above). Her birth weight was 1810 g.

The patient was vitally stable after the cesarean section and was transferred to the cardiac surgery intensive care unit (CSICU) right after the delivery with no complications.

On postoperative day 1, she was on high-flow nasal oxygen with an oxygen saturation of 80%, as well as pulmonary vasodilators. She was also on prophylactic anticoagulation. Her uterus was well contracted. She had normal lochia. She developed acute urinary retention and a new catheter was inserted. No other complications were noted and she was discharged on postoperative day 7.

In her postoperative third week, she presented to the emergency room (ER) with worsening dyspnea and an episode of pre-syncope the night prior. Chest X-ray was normal. The patient was non-compliant with her medications and was discharged after receiving 5 liters of oxygen via nasal cannula.

In her postoperative fifth week, she received the COVID-19 vaccine and presented to the ER with a fever and chills. Her oxygen saturation dropped to 70%. Again, she was managed on 5 liters of oxygen via nasal cannula and discharged after reaching her target saturation of greater than 88%.

In her postoperative sixth week, she was counseled on contraception. She had a hormonal progesterone implant inserted in her left arm as a form of contraception and it is effective for 3 years. She did not have any other complaints.

## Discussion

ES is a condition caused by an untreated congenital cardiac defect leading to a long-standing left-to-right shunt that causes pulmonary hypertension, reversal of blood flow to a right-to-left shunt, and cyanosis. ES is very rare among pregnant women with an incidence of 3% [3].

Pregnant women with ES develop volume overload due to the syndrome as well as the physiological response of pregnancy. Around 50% of circulating blood volume increases during the first 30 weeks of pregnancy, and then reaches a plateau [4]. As a result, ES patients are typically scheduled for an elective cesarean section around 32 weeks.

The decrease in systemic vascular resistance in pregnancy also increases the right-to-left shunt and therefore causes cyanosis and left ventricular dysfunction. ES secondary to atrial septal defect (ASD) has less of a mortality rate than a ventricular septal defect with it being 44% [5]. The causes of death mainly are due to thromboembolism, hypovolemia, and preeclampsia. However, our patient had no complications post-surgery.

Serial ultrasounds are recommended in ES patients due to the possibility of intrauterine growth restriction, which can affect up to 30% of fetuses [6]. In addition, there is a 50% chance of preterm delivery in ES patients [6]. In any etiology of cyanotic heart disease, fetal prognosis corresponds well with maternal hematocrit, and a successful pregnancy is unlikely with a hematocrit of more than 65% [7]. This is consistent with our patient, whose hematocrit was 38% and she had a positive fetal outcome.

The ideal mode of delivery for ES patients with severe PAH is still undetermined. Cesarean section decreases the chance of continuous increased cardiac output per uterine contraction, which could then worsen heart failure [2]. Patients with ES will deliver prematurely, and the induction process may strain their cardiac functions. Epidural anesthesia is preferred because it has a minimal effect on systemic blood pressure [2]. In a case series conducted in China, six out of nine pregnant women underwent general anesthesia for their cesarean section. This is due to the fact that general anesthesia allows for the early initiation of thromboprophylaxis [8]. However, our patient underwent a cesarean section with an epidural rather than general anesthesia and it had a favorable outcome.

Maternal mortality in pregnant women having ES with severe PAH is around 30-50% [9] and can even reach up to 65% if the patient has a cesarean section [10]. Close monitoring of these patients is required. ES with severe PAH requires a central venous catheter or right-sided catheterization or arterial line to monitor cardiac function. However, during pregnancy, invasive techniques are not recommended. Therefore, weekly or biweekly appointments are required for the care of the mother as well as the infant. Our patient was followed up weekly postpartum and had no active complaints.

Our patient had multiple antenatal visits during her pregnancy. She had ES due to ASD with severe PAH, and she still managed to have no complications during or post-cesarean section delivering a healthy female infant.

## Conclusions

In conclusion, ES is a very rare yet serious condition in pregnancy. Studies show a high maternal mortality rate in such cases, as well as a bad prognosis for the infant. Termination of pregnancy in the first trimester is recommended. However, the outcome for our case was positive for both infant and mother. The proper care of the multidisciplinary team and thorough monitoring aided in the success of this case.
